# Liver Transplantation for Intrahepatic Cholangiocarcinoma After Chemotherapy and Radioembolization: An Intention-To-Treat Study

**DOI:** 10.3389/ti.2024.13641

**Published:** 2024-10-31

**Authors:** Marianna Maspero, Carlo Sposito, Marco A. Bongini, Tommaso Cascella, Maria Flores, Marco Maccauro, Carlo Chiesa, Monica Niger, Filippo Pietrantonio, Giuseppe Leoncini, Valentina Bellia, Sherrie Bhoori, Vincenzo Mazzaferro

**Affiliations:** ^1^ HPB Surgery, Hepatology and Liver Transplantation Unit, Fondazione IRCCS Istituto Nazionale Tumori, Milan, Italy; ^2^ Department of Oncology and Hemato-Oncology, University of Milan, Milan, Italy; ^3^ Interventional Radiology, Fondazione IRCCS Istituto Nazionale Tumori, Milan, Italy; ^4^ Nuclear Medicine and Physics, Fondazione IRCCS Istituto Nazionale Tumori, Milan, Italy; ^5^ Medical Oncology, Fondazione IRCCS Istituto Nazionale Tumori, Milan, Italy; ^6^ Pathology, Fondazione IRCCS Istituto Nazionale Tumori, Milan, Italy

**Keywords:** biliary tract cancers, downstaging, gemcitabine-cisplatin, Yttrium-90, tare

## Abstract

Liver transplantation (LT) is a potentially curative experimental treatment for unresectable intrahepatic cholangiocarcinoma (iCC). Pre-transplant downstaging may help defining tumor aggressiveness and drive patient selection. We report the preliminary results of LT for liver-limited unresectable iCC after sequential downstaging with systemic chemotherapy and radioembolization (SYS-TARE). In case of sustained disease stability after SYS-TARE, patients underwent surgical nodal sampling and, if negative, were listed for LT. In this study, 13 patients with unresectable iCC underwent downstaging with SYS-TARE. The median age was 70 years and 77% were female. All had single bulky lesions at diagnosis. After SYS-TARE, 9 (69%) dropped out: 3 due to progressive disease after TARE with no response to second-line, 4 due to extrahepatic disease development and 2 due to positive nodal disease at pre-listing abdominal exploration. The median OS after dropout was 11.5 months. Four (31%) were successfully listed and transplanted. At pathology, viable tumor ranged from 30% to less than 5%. All four patients are alive and disease-free at 73, 40, 12, and 8 months from LT. LT for unresectable iCC after downstaging with SYS-TARE appears to select suitable patients for LT, achieving optimal oncological outcomes in case of response to therapy and no lymphnodal spread.

## Graphical Abstract

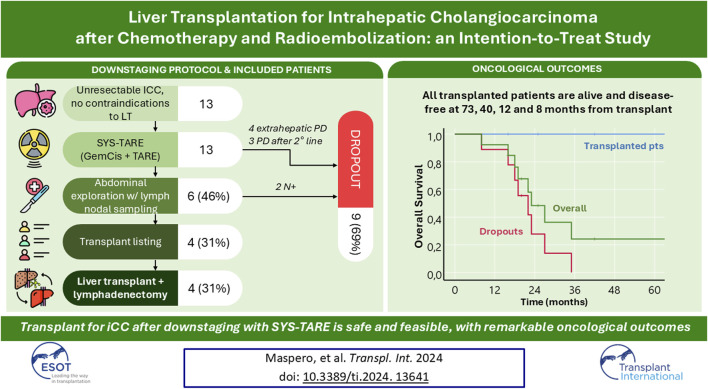

## Introduction

Intrahepatic cholangiocarcinoma (iCC) is an aggressive biliary malignancy and surgical tumor removal represents the only curative treatment option [1]. Up to 70%–80% of patients with iCC are however unresectable at diagnosis, and the median overall survival without surgery is around 18 months, with less than 10% of patients being alive at 5-year [2]. The first-line therapeutic option for unresectable iCC is systemic therapy with gemcitabine + cisplatin, in combination with durvalumab as per the recently published TOPAZ-1 trial [3, 4]. Locoregional treatment may be used in combination with systemic therapy to improve response rates and increase conversion to resection. The phase II MISPHEC trial evaluating transarterial radioembolization (TARE) plus chemotherapy as first-line treatment of locally advanced iCC suggested that this was an effective strategy, but survival without surgery remains dismal [5].

Liver transplantation (LT) expands the conventional margins of liver resection and represents an alternative curative-intent option for patients with unresectable disease [6]. However, with the exception of cirrhotic patients with small tumors (≤2 cm), LT alone does not confer a significant survival advantage in iCC [7]. Conversely, patients with unresectable iCC that respond to downstaging seem to be the best candidates for LT. In a recent experience from Houston Methodist, they reported a 5-year survival of 83% for six highly selected cases with locally advanced iCC who were transplanted after intensive neoadjuvant therapy [8]. Their experience was updated in 2022, with the report of 32 listed patients and 18 transplants with a 5-year overall survival of 57% [9].

Since 2018, our Center has implemented an intention-to-treat strategy for unresectable iCC that draws from those experiences and combines them in a multistep sequential protocol of local and systemic treatment to select LT candidates considered suitable candidates after multidisciplinary (MDT) assessment. The protocol takes advantage of a consistent experience with radioembolization as a mean to deliver radiation therapy to liver tumors. Here we report the intention-to-treat outcomes of the first thirteen cases, of which four (31%) were successfully transplanted.

## Patients and Methods

### Combined Systemic Therapy–Radioembolization (SYS-TARE) Protocol

**FIGURE 1:**
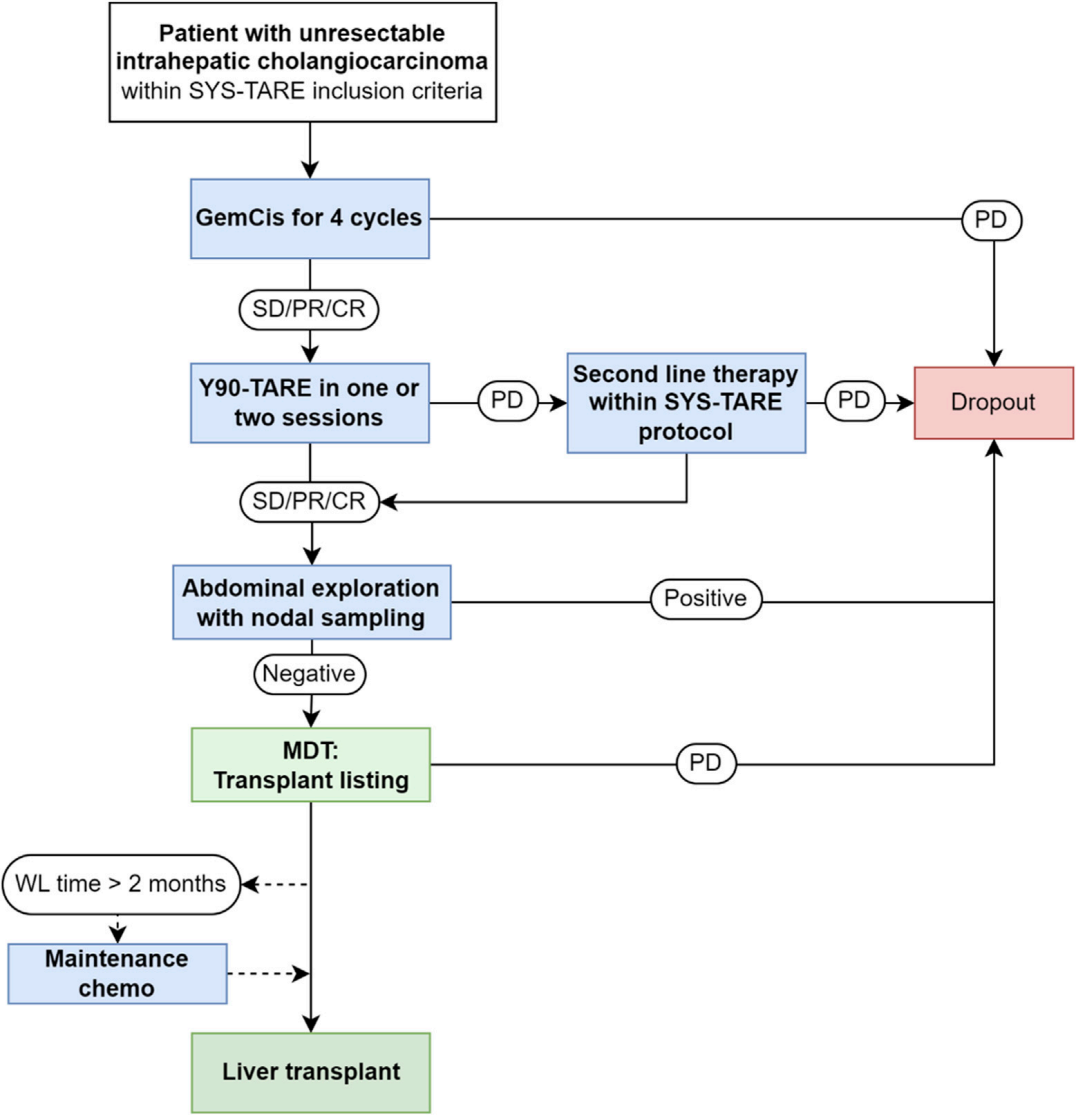
Neoadjuvant combined systemic therapy and radioembolization (SYS-TARE) protocol for unresectable intrahepatic. iCC, intrahepatic cholangiocarcinoma; GemCis, gemcitabine + cisplatin; SD, stable disease; PR, partial response; CR, complete response; PD, progressive disease; MDT, multidisciplinary team; WL, waitlist; CT, chemotherapy. Second-line chemotherapy was indicated according to standard of care, preferably with targeted therapy if actionable mutations were present at next-generation sequencing analysis. Maintenance chemotherapy included additional cycles of GemCis until transplant or disease progression.

The flowchart of the protocol applied to patients with unresectable iCC with liver-only tumor presentation and no absolute contraindications to LT is reported in Figure 1. Inclusion criteria for the protocol were: 1) Histologically proven mass-forming iCC with a single measurable lesion with or without associated peritumoral satellites; macroscopic vascular encasement was allowed as long as tumor thrombosis was excluded and the extent of tumor vascular encasement was limited to the intrahepatic portion of portal/hepatic veins; 2) Unresectable disease due to tumor location or underlying liver disease; 3) Age between 18 and 70 years; 4) No lymphatic or extrahepatic spread; 5) Performance status 0-1; 7) Written informed consent. Exclusion criteria were: 1) Multifocal iCC involving multiple segments; 2) Macroscopic vascular thrombosis/tumor invasion; 3) Prior resected extrahepatic tumor spread; 4) Concomitant malignancies or history of other malignancies in the previous 5 years; 5) Non-oncological contraindications to LT.

Enrolled patients underwent a sequential downstaging treatment with 4 cycles of gemcitabine + cisplatin, followed by TARE with Yttrium^90^ glass microspheres (Therasphere, Boston Scientific, Marlborough, MA) in one or two sessions.

### Chemotherapy, Radioembolization and Evaluation of Response

Chemotherapy was started after pathology confirmation of intrahepatic cholangiocarcionoma and assessment of non-resecability by an experienced hepato-biliary surgical team. Chemotherapy consisted of at least four cycles of gemcitabine (1000 mg/m^2^) and cisplatin (25 mg/m^2^) administered intravenously on day 1 and day 8 of a 21-day cycle, as per standard of care.

Trans-arterial radioembolization (TARE) was performed, as previously described [10]: a simulation of treatment was performed by the injection of ^99^Tc-MAA into the hepatic arterial vasculature reproducing Y^90^ microspheres distribution, in order to estimate the degree of lung shunt and/or extrahepatic deposition and tumor uptake by means of planar and SPECT scintigrams. The dose calculation was individualized according to ^99^mTc-MAA SPECT voxel dosimetry [11]. Patients were treated on average with 2.8 million of microspheres per GBq. The treatment was performed two to 3 weeks after the simulation, by the injection of glass microspheres loaded with ^90^Yttrium on the day of admission. Before injection, patients were given 2 g of cefazoline intravenously. After TARE, patients were hospitalized for 48 h for clinical observation.

All patients underwent restaging with CT scan, FDG-PET and tumor markers after four cycles of chemotherapy, then 1 month after TARE, then every 2 months. Follow up continued in the same manner every 2 months while on the transplant waitlist. Response to SYS-TARE was evaluated with CT scan according to RECIST criteria [12] and Choi criteria [13], and FDG-PET. In particular, response according to Choi was calculated by assessing the change in density of the most vascularized and/or representative slice of the entire lesion during an arterial phase at baseline and after treatment. In case of partial response or stable disease according to the previous radiological/metabolic criteria, as well as a comparable CA19-9 decrease/stability for at least 4 months, the patients underwent a surgical exploration of the abdomen (either laparotomic or laparoscopic) to determine disease burden with intraoperative ultrasound, peritoneal exploration and washing and lymph nodal assessment.

Assessment of lymphatic spread involved nodal sampling of stations 8 and 12 in absence of clinically suspicious nodes, aiming for a minimum of 5 lymph nodes for adequate assessment; in patients with suspicious nodes at pre-op imaging, lymphadenectomy of suspicious nodes was performed in addition to stations 8 and 12. In clinically N0 patients, the dissection was carried out until sufficient tissue was retrieved to assess the minimum number of required nodes. In patients with suspicious nodes, the dissection was carried out until all radiologically suspicious nodes were excised. In case of persistence of suspicious lymph nodes at post-surgical exploration imaging, a re-exploration was performed. Cytology from peritoneal washing was also carried out in all cases: 250 mL of saline were injected in the abdominal cavity and retrieved after putting the patient in Trendelenburg position. After having excluded the presence of extrahepatic disease, a formal assessment of transplant eligibility was conducted during the dedicated MDT and in case of no general contraindications the patient was listed for LT. No specific priority was assigned in case of LT listing even though reassessment of priority was planned every 2 months while on active list.

At whichever stage of the process, in case of intrahepatic disease progression second line therapy was allowed preferably with targeted therapy in case of presence of actionable mutations at next-generation sequencing (NGS) analysis and waitlisting reconsidered only in case of partial response for at least 4 months. Disease stability was assessed every 2 months while on the waiting list. In case of waiting time longer than 2 months, additional cycles of gemcitabine and cisplatin were allowed. In case of extrahepatic progression or positive nodal sampling, the patient dropped out of the protocol. LT was carried out with grafts procured from deceased donors according to standard practice. During total hepatectomy, *en bloc* lymphadenectomy of stations 8, 9, 11p, 12a, 12b, 12p, and 13, if not previously removed, was performed in all patients [14].

### Statistical Analysis

Descriptive variables were calculated for the overall cohort: categorical variables were expressed as number (percentage), while numerical variables as median (interquartile range). Median follow-up was calculated with the inverse Kaplan Meier method. Overall survival (OS) was calculated using the Kaplan Meier method, with censoring at death or last follow up: OS was calculated from diagnosis and from last treatment within the SYS-TARE protocol for the overall cohort; from dropout for patients who exited the SYS-TARE protocol; and from transplant for patients who were successfully transplanted. Disease-free survival, defined as the interval between LT and iCC recurrence, was calculated for transplanted patients using the Kaplan Meier method. All analyses were conducted using IBM SPSS Statistics for Windows, version 26 (IBM Corp., Armonk, N.Y., United States).

## Results

Since 2018, thirteen patients were enrolled into the protocol. Their characteristics are reported in Table 1. The median age was 60 years, and the majority (77%) were female. Four (31%) patients had iCC arising on normal liver, 7 (57%) on metabolic-associated steato-hepatitis, 1 (7%) on Wilson’s disease and 1 (7%) on hepatitis B virus-associated chronic liver disease. All except one had a single lesion at diagnosis, with a median diameter of 100 mm. Non-resectability was due to central location with hepatic outflow encasement in 12 patients, and to tumor location and underlying Wilson’s disease in one case. Patients received on average 6.5 cycles of chemo for a median time of 4.5 months. All patients underwent TARE with ^99^mTc-MAA SPECT voxel dosimetry.

**TABLE 1:** Characteristics of the overall cohort.

Variable	Overall cohort (n = 13)
Age at diagnosis (years)	60 (55–67.5)
Sex *Female* *Male*	11 (77%) 3 (23%)
Liver status *Healthy* *MASLD* *HBV* *Wilson’s disease*	4 (31%) 7 (56%) 1 (7%) 1 (7%)
Comorbidities *Hypertension* *Dyslipidemia* *Smoking habit* *Obesity (BMI ≥ 30)* *COPD*	4 (31%) 3 (23%) 3 (23%) 2 (15%) 1 (7%)
Number of lesions at diagnosis	1 (1)
Size of largest lesion at diagnosis (mm)	100 (62.5–117)
CEA at diagnosis (ng/mL) CA19-9 at diagnosis (U/mL)	3 (1.5–4) 48 (29–189)
Number of total GemCis cycles	7 (4–11)
Number of TARE *One* *Two*	7 (56%) 6 (43%)
Mean dose to the lesion (Gy) *First TARE* *Second TARE*	311 (206–629) 359 (89–833)
Dropout	9 (69%)
Time from TARE to dropout (months)	5 (2.5–6.5)
Median follow up (months)	43 (30–81)
Deaths	8 (61.5%)
Median survival after dropout	11.5 (6.5–14.5)

Data is number (percentage) and median (interquartile range).

MASLD, metabolic-associated steatotic liver disease; HBV, hepatitis B virus; BMI, body mass index; COPD, chronic obstructive pulmonary disease; GemCis, gemcitabine + cisplatin; TARE, radioembolization; Gy, gray.

**FIGURE 2:**
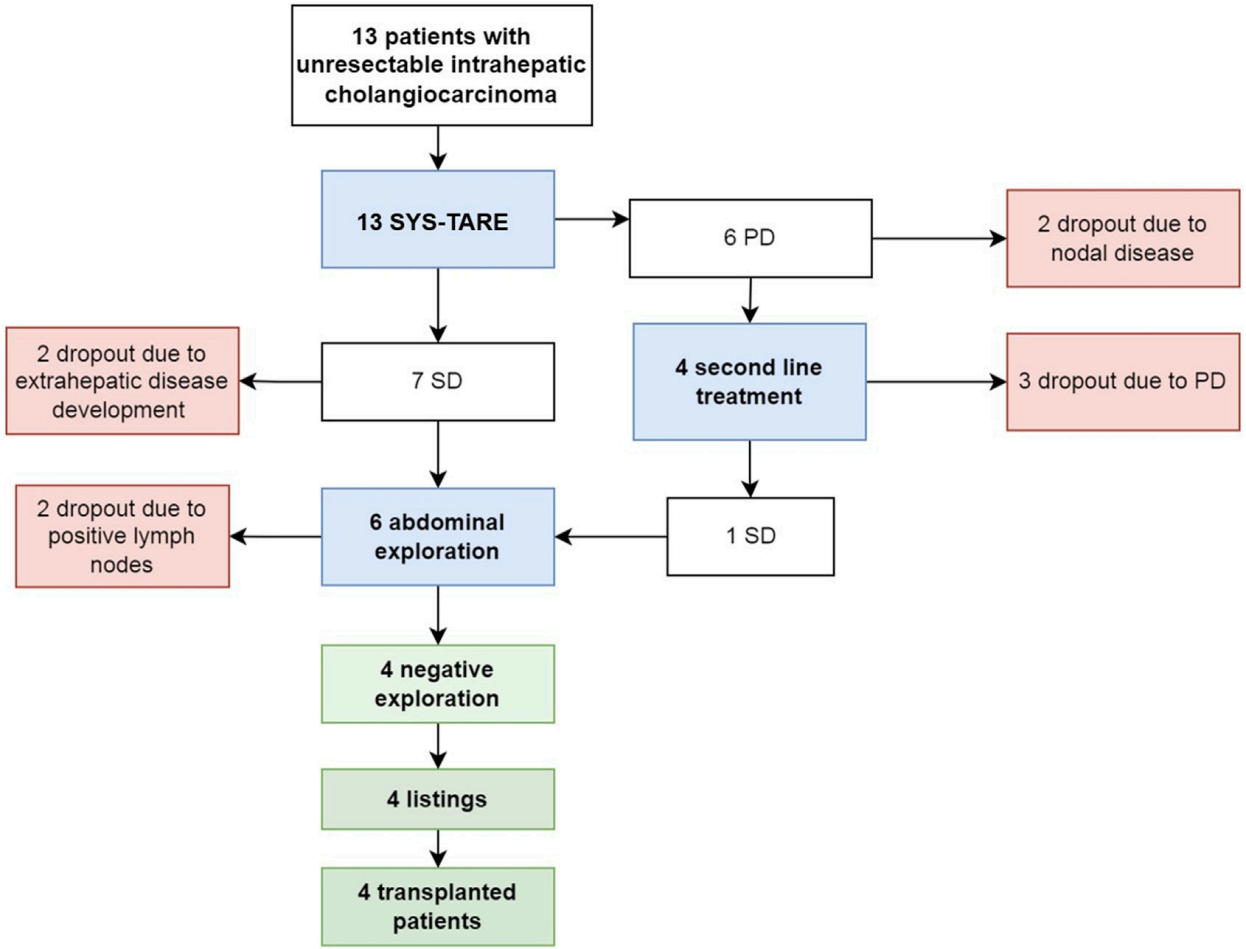
Flowchart summarizing treatment allocation and evolution of 13 unresectable mass-forming iCC, according to tumor response after combined chemotherapy and radioembolization.

The median dose delivered to the lesion during TARE was not significantly higher in patients who subsequently underwent transplant (626 Gy, IQR 427–1462) than patients who dropped (224 Gy, IQR 159–316), p = 0.059. Dropout occurred in 9 (69%) cases after a median of 5 months, while four (31%) patients were listed and transplanted (Figure 2). Dropout was due to progression of disease (PD) after TARE in 4 cases, after second line treatment in 3 cases, and due to tumor spread in the hilar lymphnodes at abdominal exploration (N+) in 2 cases.

### Transplanted Patients

**FIGURE 3:**
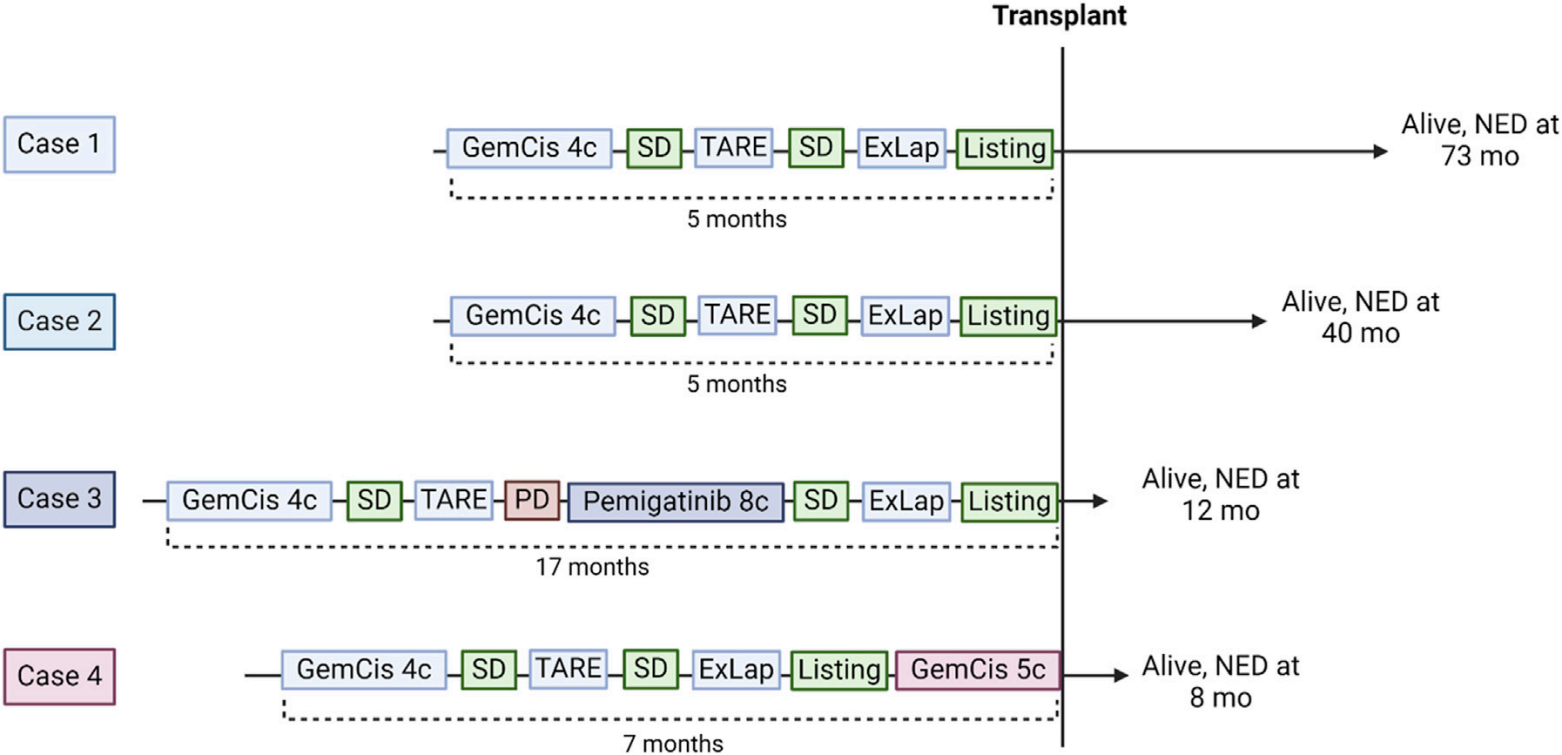
Downstaging sequences of the 4 responding patients who underwent liver transplantation. GemCis, gemcitabine + cisplatin; C, cycles; SD, stable disease; TARE, transarterial radioembolization; ExLap, exploratory laparotomy/laparoscopy; PD, progressive disease; NED, no evidence of disease.

Four out of 13 (31%) patients were successfully downstaged and transplanted within the protocol. Their neoadjuvant treatment sequences are summarized in Figure 3. Two patients underwent the SYS-TARE sequence, had sustained disease stability, were listed for LT and transplanted within 2 months from listing. One patient had sustained disease stability after the SYS-TARE sequence but received 5 additional cycles of gemcitabine + cisplatin because of increased waiting time. One patient had an initial disease stability but developed intrahepatic disease progression after TARE with a single subcentimetric lesion. The patient underwent NGS analysis where an actional FGFR2 mutation was found. The MDT decided to proceed with a second-line treatment with the FGFR2 inhibitor pemigatinib, to which the patient had a partial response, leading to listing after 8 cycles. All patients had an uneventful negative abdominal exploration (laparoscopic in 3 cases, laparotomic in 1). Two out of four patients required two TARE sessions due to large centrohepatic lesions with bilobar feeding arteries. The average dose/sphere delivered of radiotherapy was 626Gy (IQR 427–1462) at first TARE and 495 Gy (40–951) at second TARE. Neither severe adverse event or dose reduction related to chemotherapy or TARE treatment were registered. The best response obtained before listing was stable disease (SD) in all patients according to RECIST criteria, and partial response (PR) in all patients according to Choi criteria with a median decrease of tumor density of 52.5%.

Peri- and post-transplant characteristics are shown in Table 2. The median intervals from diagnosis to listing and transplant were 6 and 9 months, respectively. All patients had good liver function (median MELD 7), CEA <10 ng/mL and CA 19-9 < 100 U/mL both at listing and at transplant. The median time on waitlist was 57 days, while the median time between the last treatment and LT was 36 days. All patients received full grafts from deceased donors (3 after brain death, 1 after cardiac death). The donor-risk index was 1,0 (range 1.1–2.2). All donors were extended criteria donors for at least one characteristic: two were older than 70 years, one had a ICU stay longer than 10 days, and one was a donor after cardiac death (DCD). The graft from the DCD donor underwent hypothermic oxygenated machine perfusion for 2 h. The median OR time was 10 h. Two cases required removal of the native vena cava to ensure oncological radicality due to lesions located near the hepatocaval confluence, while the other two received a piggy-back implantation technique. No intraoperative complications occurred.

**TABLE 2:** Characteristics of transplanted patients at listing, transplant, and follow-up.

	Overall (n = 4)	Case 1	Case 2	Case 3	Case 4
Interval between diagnosis and LT listing (months)	Median 6	5	5	17	7
MELD-NA at listing	Median 7	7	6	8	7
Presence of viable tumor at listing (DWI or PET)		—	Yes	—	Yes
RECIST response at listing	100% SD	SD	SD	SD	SD
Choi response at listing Decrease in tumor density from baseline to listing	100% PR Median 52.5%	PR 47%	PR 35%	PR 58%	PR 73%
CEA at listing CA19-9 at listing	Median 3.9 Median 40	4.05 22	3.2 38.4	6.63 46.9	3.8 42
Time on waitlist (days)	Median 57	49	2	65	127
Interval diagnosis and LT (months)	Median 10.5	10	5.5	19	11
Interval last treatment and LT (days)	Median 36	59	58	5	14
CEA at LT (mg/dL) CA19-9 at LT (mg/dL)	Median 3.3 Median 43	3.35 17.7	2.7 41.5	8.5 75.7	3.4 44.5
MELD-Na at LT	Median 7	6	7	7	7
LT duration (hours)	Median 10	13	11	9	9
Venovenous bypass	2/4 (50%)	Yes	No	Yes	No
Final pathology	4/4 iCC	iCC	iCC	iCC	iCC
TNM staging T1aN0 T2N0	2/4 (50%) 2/4 (50%)	ypT2N0	ypT1aN0	ypT1aN0	ypT2N0
Number of lesions	Median 1	1	1	2	1
Size of lesions (mm)	Median 74	72	23	76	84
% viable tumor at final pathology	Median 25%	30%	30%	<5%	20%
Grading	3/4 (75%) G3 1/4 (25%) G2	G3	G3	G3	G2
Lymphovascular invasion	2/4 (50%)	Present	Absent	Absent	Present
Perineural invasion	2/4 (50%)	Present	Absent	Absent	Present
Number of metastatic/retrieved lymph nodes	0/8	0/7	0/9	0/10	0/6
LOS (days)	Median 11.5	12	11	44	10
Postoperative complications	Major complications 2/4 (50%)	Pleural effusion CD II	Yes, partial hepatic artery thrombosis CD IIIa CCI 45.4	Yes Biliary stenosis, hemoperitoneum, duodenal fistula CD IIIb CCI 73.7	No
Follow-up (months)	Median 26	73	40	12	8
Recurrence	0/4	No	No	No	No
Alive, NED at latest follow up	4/4	Yes	Yes	Yes	Yes

Data is number (percentage) and median (interquartile range). LT, liver transplant; CRLM, colorectal liver metastases; T-bil, total bilirubin; MELD-Na, model for end stage liver disease–sodium; DBD, donation after brain death; LDLT, living donor liver transplant; CIT, cold ischemia time; WIT, warm ischemia time; LOS, length of stay; CD, clavien dindo grade; CCI: comprehensive complication index; NED, no evidence of disease.

The median length of hospital stay was 11.5 days. Two patients experienced major complications within 90 days: one developed partial hepatic artery thrombosis requiring stent placement (comprehensive complication index, CCI 45.4); another developed a kinking and leak of the biliary anastomosis, requiring reoperation and hepatojejunostomy, followed by duodenal leak requiring pancreatoduodenectomy and two further operations for hemoperitoneum and eventration (CCI 73.7).

**FIGURE 4:**
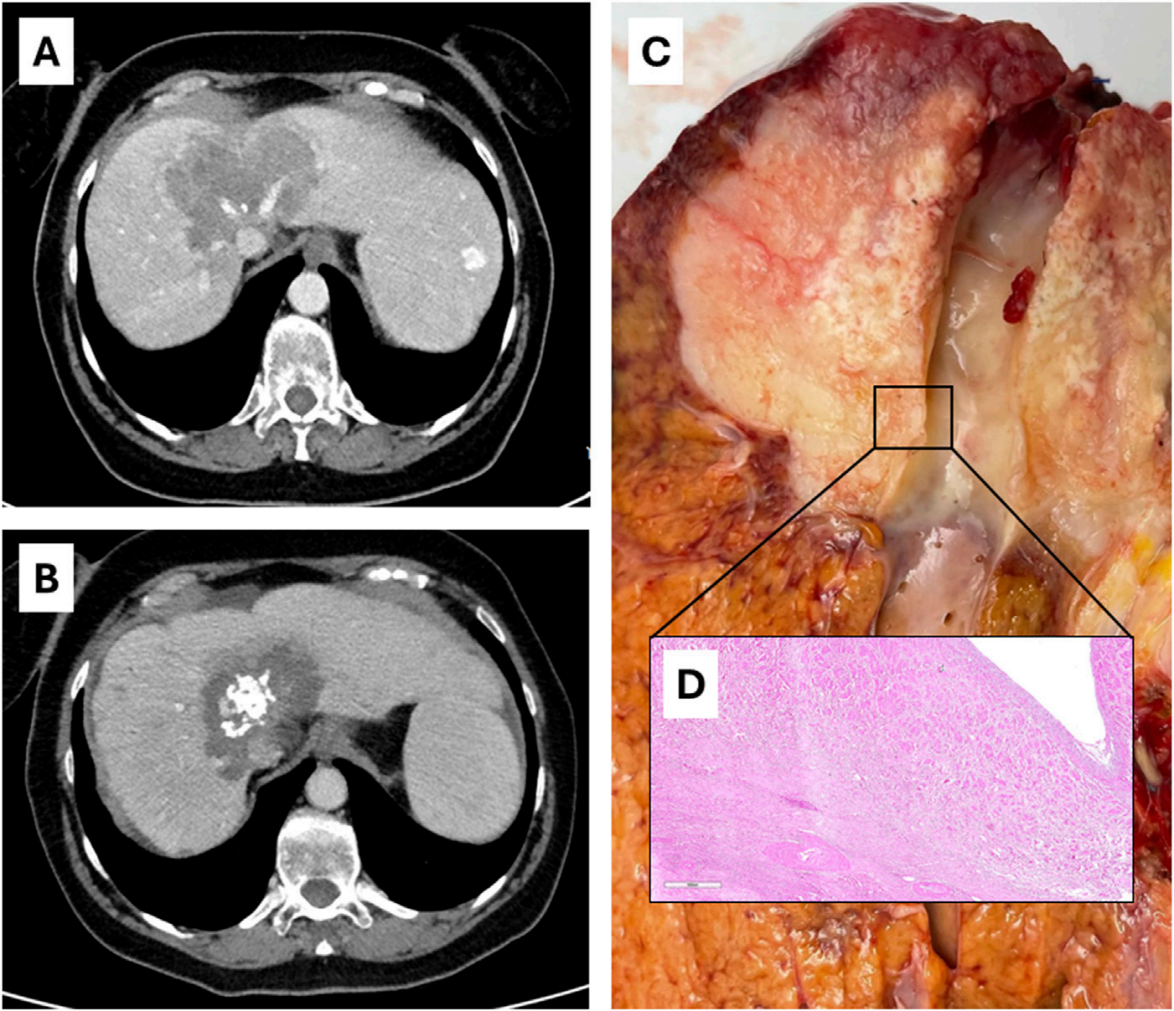
Case 3 of Table 2: radiological [**(A)**, before therapy; **(B)**, after SYS-TARE], *ex vivo* **(C)** and histological **(D)** appearance of hepatic vein encasement without intimal penetration.

At final pathology, in all patients the diagnosis of mass-forming iCC was confirmed with various degrees of response to neoadjuvant therapy, with residual viable tissue ranging from 30% to less than 5%. No patient had satellitosis nor invasion of major intrahepatic vessels. In all cases of hepatic vein encasement, histology confirmed that the tumor did not invade the intima (Figure 4). With a median of 8 retrieved lymph nodes, all patients were N0. After MDT discussion, no patient underwent post-transplant adjuvant therapy. Given the lack of disease recurrence, no NGS analysis was performed on the other three patients.

The post-transplant immunosuppressive regimen included steroids and tacrolimus. All patients stopped steroids within 1 months from transplant. Currently, two patients are on tacrolimus alone, while two patients are on a combination of tacrolimus and everolimus due to monotherapy intolerance.

### Long-Term Follow Up

**FIGURE 5:**
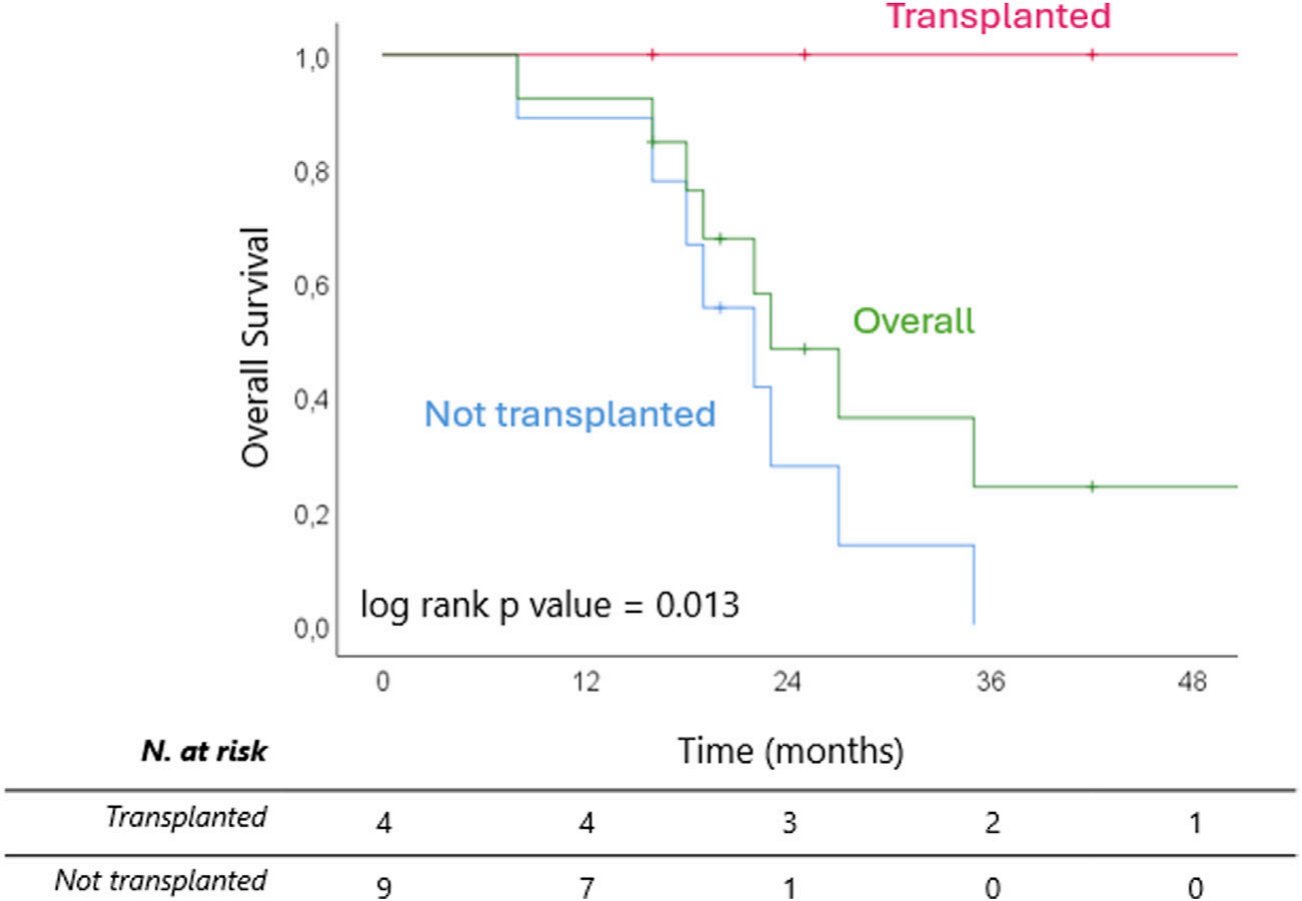
Kaplan Meier curves of overall survival from completion of SYS-TARE (i.e., last TARE within protocol) for transplanted and non-transplanted patients.

After a median follow up of 45 (IQR 32–83) months from diagnosis, 8 patients (61.5%) are dead, all belonging to the non-transplanted cohort. Median OS from diagnosis was 33 (IQR 26–42) months overall and 29 (IQR 23–33) months for the non-transplanted cohort (Figure 5). The median OS from last treatment within the SYS-TARE combo was 18 (12–35) months overall and 17 (12–19) months for the non-transplanted cohort. The median OS after dropout was 11.5 (6-5–14.5) months with 4 patients surviving for at least 1 year. All deaths were cancer-related due to disease progression. Of the two patients in the non-transplanted cohort who are still alive, one is undergoing hospice care, while the other is in stable disease after a rechallenge with GemCis with the addition of durvalumab. All transplanted patients are alive and disease-free at 73, 40, 12, and 8 months from LT.

## Discussion

In the presented case series of 13 patients, the preliminary results of liver transplantation for unresectable intrahepatic cholangiocarcinoma after sequential downstaging with chemotherapy and radioembolization (SYS-TARE) is reported. Out of thirteen patients, four (31%) were successfully transplanted after neoadjuvant SYS-TARE, with one requiring an additional line with targeted therapy due to progression after TARE. The median intention-to treat survival of the presented consecutive series was 33 (IQR 26–42) months, which compares favourably with the median OS of 12.8 (11.1–14.0) months observed with the current standard of care [3]. All patients who had a sustained response up to transplant are alive with no evidence of recurrence after a median of almost 2 years of follow up.

The rationale for our prospective protocol draws from the pioneering experiences with LT for ICC from several contexts [8, 9, 15], as well as from the advances in the systemic and locoregional treatment of unresectable iCC [16]. It is increasingly evident that LT for iCC can offer a significant survival benefit, differently from what was previously thought, if the key principles residing in pre-transplant tumor response to combined chemo-radiation treatments and consequent patient selection are respected. After all, therapeutic efficacy and depth of response could be considered valid surrogates of tumor biology in iCC, as increasingly demonstrated in other transplant oncology indications, such as LT for colorectal liver metastases, HCC and perihilar cholangiocarcinoma after multimodal downstaging protocols [8, 17].

Our patient population was carefully selected according to pre-determined criteria combined with dynamic assessment of tumor response to treatment. Tumor presentation was also considered, as patients with multifocal disease were excluded as associated with unfavorable biology, while vein encasement by single bulky lesions was not considered as a contraindication for LT consideration. As a matter of fact, all but one patient had single lesions at baseline, with no satellitosis and no clinically suspicious lymph nodes. All patients were not jaundiced and, in such condition, CA19-9 had to be below 100 U/mL for LT consideration. Those characteristics are similar to those reported by McMillan et al. [9] and confirm that tumor size in iCC on non-cirrhotic liver has a relatively negligible impact on prognosis, as suggested by both transplant and non-transplant series [15, 18]. Chronic liver disease in our series was minor, mainly related to metabolic syndrome without untreatable comorbidities, except one patient who had Wilson’s disease which was cured with LT.

The presented protocol differs from previously published experiences due to its intention-to-transplant design. In contrast with others [8], in which patients were selected for LT consideration among those responding to non-systematic neoadjuvant treatments, our patients entered the protocol from first referral with a specific downstaging-to-transplant aim. This accounts for a dropout rate of 69% before LT listing, mostly occurring between TARE and abdominal exploration due to intrahepatic progression. The test of time between TARE and listing seems therefore to be important for accurate patient selection. Conversely, the added radioembolization in our and other series seems to guarantee a surprisingly long survival despite progression and dropout from LT consideration. The consequent suggestion to expedite patient listing in case of objective radiological response to TARE in iCC needs further confirmation and more extended follow-up.

The present study is not the first report of LT for iCC after TARE: Gruttadauria et al. [19], in 2021, reported two patients who received TARE as neoadjuvant therapy before LT for iCC (although one had a mixed hepatocellular-cholangiocarcinoma). The presented protocol combined first-line chemotherapy with TARE as a source of radiotherapy in single bulky unresectable iCC and proved to be effective in terms of pathological response and patient outcomes. This combination achieved at least 70% of tumor response in all patients, up to over 95% in one patient. Although the superiority of the combination of radiation therapy + chemotherapy versus chemotherapy alone in unresectable iCC is not supported by randomized controlled trials, we believe that the available retrospective and prospective evidence showing consistent benefits in terms of local disease control [5, 20–22], as well as high tolerability in the non-cirrhotic setting [23], strongly supports the use of this combination as a neoadjuvant strategy before liver transplantation and resection.

The rationale for the combination of systemic therapy and TARE in iCC is even stronger now that the new standard of care for the treatment of unresectable iCC adds to the GemCis scheme the immune checkpoint inhibitor (ICI) durvalumab [3]. The synergistic effect of ICI and radiation is supported by mechanistic notions as well as clinical evidence [24–26], and has been demonstrated also after TARE [27]. The abscopal effect of radiation therapy on the antitumoral immune response is well known, and this effect is even more robust when combined with ICI. For this reason, our protocol has been updated to include GemCis + durvalumab followed by TARE with the aim of achieving even a more profound and sustained local control in downstaging-to-transplant for iCC [25]. For the time being, we have decided to offer chemotherapy, which is the standard of care treatment for advanced iCC, before TARE, however if the rationale of this downstaging strategy is confirmed, it could be considered to offer TARE first to boost the effects of the subsequent chemo-immunotherapy.

Another hint of flexibility in neoadjuvant approach to iCC is targeted therapy allowed as second-line, as per standard of care. The patient who underwent LT after second-line FGFR2 inhibitors demonstrated the most profound pathological tumor response, with less than 5% viable tumor tissue in the hepatectomy specimen. This is in line with previous experiences in the LT setting [9]. Accordingly. more systematic tumor profiling in patients entering protocols of ore-LT downstaging needs to be further investigated.

With respect to tumor response, pre-transplant radiological assessment of response to SYS-TARE was poorly encapsulated by RECIST criteria, that classified all pre-LT observed responses as stable disease, while explant histology demonstrated more relevant effects. Consequently, RECIST criteria may not be appropriate for evaluation of iCC in the setting of neoadjuvant treatments, and similar considerations may be made regarding modified RECIST (mRECIST) criteria, as it is challenging to give an mRECIST evaluation of lesions with an hyperenhancing border as is often the case with iCC. In our experience, Choi criteria appear to have a higher correlation with pathological response [13, 28].

All patients who were made eligible to LT underwent abdominal exploration with nodal sampling before listing. The invasiveness of such surgery, especially in case of hilar lymphadenectomy, may be questioned. However, occult lymph node metastases in iCC occur in 24%–40% of T1-T2 tumors [29], and are a significant prognostic factor after resection [30]. Accordingly, the preliminary assessment of at least stations 8 and 12 are deemed crucial in patient selection as those stations cover >80% of possible lymph nodal metastatic sites in iCC [31]. Two out of six patients (33%) who underwent abdominal exploration were excluded due to positive nodal spread that was not detect with imaging or FDG-PET. Differently from other experiences [9], our protocol did not include adjuvant therapy and no patient was deemed eligible to adjuvant therapy due to unexpected nodal positivity or high-risk features at pathology.

Finally, a mention should be given to the widespread concern that expanding the indications for transplant oncology will result in an unbearable pressure on the donor pool. Our experience, similarly to others before ours, does not seem to support this concern. First, the cohort of patients with iCC who fulfills the criteria for transplantation with acceptable 5-year survival represent a minority of patients for an already rare disease. Secondly, as shown by our median donor risk index, most of these patients were transplanted with marginal grafts with excellent long-term functional results. For these reasons, it seems unlikely that the inclusion of carefully selected patients with iCC into the standard indications for LT will result in an unacceptable increase in transplant candidates in most local scenarios. Median time on waitlist in our cohort was around 2 months, which we recognize may not be as easily achievable in different scenarios. Given the excellent depth of response in the patients who eventually made it to LT and the lack of dropouts during the waiting period, it can however be speculated that carefully selected patients may withstand longer waiting periods, especially if they can continue systemic treatment in the meantime.

This study has several limitations. It is a monocentric experience with a small sample size. There is no control group, thus the survival benefit of LT in this specific population can only be inferred using comparisons from the literature. Chemotherapy was performed according to the standard of care at the time (i.e., GemCis), which is not the standard anymore. Finally, two of the four patients who underwent transplant have less than 2 years of follow-up from LT.

In conclusion, the intention-to-treat results of this series of 13 patients with unresectable iCC who underwent of neoadjuvant SYS-TARE suggest that this combination may results in sustained response rates that could be considered sufficient to offer LT with excellent survival, if associated to pre-transplant abdominal exploration excluding nodal disease. Post-transplant outcomes in this setting compare favourably with previous reports offering non-transplant options and with the patients who continued follow-up without LT. Further prospective studies with larger sample sizes and longer follow-up are needed to confirm our findings.

## Data Availability

The raw data supporting the conclusions of this article will be made available by the authors, without undue reservation.

## Abbreviations

LT, liver transplantation; iCC, intrahepatic cholangiocarcinoma; TARE, transarterial radioembolization; MDT, multidisciplinary team; SYS, systemic therapy.
